# Application of Grape Stems Extract to Increase the Antioxidant Capacity of Whiskey

**DOI:** 10.1155/2024/7199030

**Published:** 2024-08-08

**Authors:** Anatoliy Kazak, Yurij Grishin, Joel B. Johnson, Nikolay Oleinikov, Marina Rudenko, Angela Mayorova, Anna Dorofeeva, Kristina Frolova

**Affiliations:** ^1^ Humanitarian Pedagogical Academy V.I. Vernadsky Crimean Federal University 295007, Simferopol, Russia; ^2^ Laboratory of Analytical Research, Innovative and Resource-Saving Technologies All-Russian National Research Institute of Viticulture and Winemaking “Magarach” 298600, Yalta, Russia; ^3^ School of Health Medical and Applied Sciences Central Queensland University, North Rockhampton, QLD 4701, Australia

## Abstract

The recent steady demand for products with increased antioxidant properties largely determines the direction of research in the field of creating high-quality products with increased biological value. Insufficient knowledge of the antioxidant activity and potential biological value of whiskey makes this area of research relevant and in demand. This study presents the results of an analysis of the influence of distillate aging conditions on the phenolic composition and antioxidant capacity of whiskey, obtained using coulometric, chromatographic, and amperometric methods of analysis. During the study, samples of whiskey with increased antioxidant capacity were obtained based on the use of alcoholic extracts of grape stems of the *Vitis vinifera* species. The data obtained as a result of the study can be used in the future both to improve the efficiency of methods for monitoring and regulating the circulation of alcoholic products based on the developed methodology for determining antioxidant capacity and to contribute to the creation of a new type of alcoholic products with an increased content of bioavailable biologically active substances and antioxidant capacity.

## 1. Introduction

Since ancient times, the basis of the prevailing majority of food products has been cereals and their processed products. Alcoholic drinks made from cereals are in wide demand in modern society. One of these drinks, which has gained the greatest fame and acquired the largest number of connoisseurs, is whiskey, the production process of which involves the preparation of malt based on barley, corn or rye, fermentation of malt, distillation, and aging of distillates [1, 2]. Currently, many studies have been conducted aimed at studying the antioxidant properties of products prepared from the processing of plant raw materials [3–6]. At the same time, as a result of epidemiological studies, it was found that the inclusion of aged distilled drinks in the daily diet has an unconditional positive effect on the human body during coronary heart disease, and also improves lipid metabolism [7, 8].

It has been established that antioxidant capacity is largely determined not only by the total content of water-soluble antioxidants but also by their qualitative and quantitative composition and by their ability to interact with reactive oxygen species [9, 10]. It is known that phenolic substances synthesized exclusively in plant tissues are capable of interacting with free radical molecules, acting as inhibitors of free radical reactions and suppressing the high reactivity of free radicals [11, 12].

The phenolic composition of plant tissues of wheat, as the main plant raw material used in the production of whiskey, is represented by biologically active substances, in particular phenolic acids: ferulic, vanillic, caffeic, syringic, and *p*-coumaric acids. Ferulic acid is the main and most abundant phenolic acid in whole-grain cereals [13, 14]. During the distillation of fermented and fermented malt, small amounts of volatile phenolic substances pass into the distillate, which practically does not participate in the formation of the phenolic profile of distillates aged in oak barrels. The determining factor for the phenolic profile of distillates aged in oak barrels is the use of a technological method in the production of whiskey—aging in oak barrels [15, 16]. The multifactorial nature of the aging process and, as a consequence, the degree of enrichment of distillates with phenolic substances are directly dependent on factors such as the type of oak (such as Quercus robur, Q. petraea, and Q. alba), each of which is characterized by its individual structure and fiber composition; the volume of oak barrels; and the duration of the aging process itself. The chemistry of the process of extracting phenolic substances from wood lies in the processes of hydrolysis of lignins, gallotannins, ellagitannins, and other valuable components of oak wood occurring at the interface between the liquid and solid phases [17, 18]. As studies show, the decisive role in the formation of the chemical composition, organoleptic properties, and qualities of aged distillates belongs to the compounds released into the distillate from oak wood, namely, low molecular weight phenolic compounds and tannins [19]. Oakwood serves as a source of compounds such as ellagic and carboxyellagic acids.

Conducted in vivo studies on the antioxidant capacity and biological value of phenolic substances have established that representatives of phenolic substances such as (+)-D-catechin, gallic, and ellagic acids are characterized by high antioxidant and biological activities. As a result of numerous demographic studies, significant numbers of test populations have been analyzed, and the most applicable norms for daily consumption of phenolic substances with high antioxidant capacity have been established. The average daily intake of catechins varies widely, from 77 to 182 mg/day, and varies depending on the geographical features and botanical diversity that determine the composition and structure of the respondent's diet [20]. According to Dutch researchers, the main bioavailable representative of catechins is (+)-D-catechin, the average consumption of which was about 50 mg/day [21]. The detoxifying properties of catechins are most pronounced in (+)-D-catechin, which inhibits DNA damage caused by potential food carcinogens such as heterocyclic amines. It has been established that the average daily intake of phenolic acids in the human diet is about 200 mg/day, while this value is largely averaged and depends on the individual characteristics of the diet [22]. In particular, the daily intake of hydroxybenzoic acids coming from food is estimated at 25–100 mg/day [23].

A high content of these representatives of phenolic substances characterizes one of the most widespread types of waste in the viticulture and wine industry—grape stems, which can act as a valuable source of biologically active substances and antioxidants with a phenolic structure. The fact that the grape plant belongs to the tree-like vines largely determines that the composition of structural elements, such as grape stems, is similar in a number of indicators to the composition of tree wood, including oak species used for the aging process of alcoholic beverages. Thus, such a biologically active substance as ellagic acid is found both in oak wood, such species as follows: North American white oak (*Quercus alba*) and European red oak (*Quercus robur*), and in grapes (grape stems) [24]. Ellagic acid is a low molecular weight phenolic compound, a dilactone of a phenolcarboxylic acid of a hydrolyzable group, which is a dimeric derivative of gallic acid, formed as a result of the hydrolysis of ellagitannin and geraniin. Ellagic acid has pronounced cardioprotective activity [25]. Thus, at a dose of 0.5 and 1 mg/kg, it is more effective than vitamin E at a dose of 50 mg/kg. Ellagic acid has a hypotensive effect by participating in the mechanism of the inflammatory response [26]. It has also been found to have antimutagenic [27], antioxidant [28], antiproliferative [29], fermenting inhibitory, reparative, and other types of activity [30]. At the same time, in a drink such as whiskey, according to J.M. Landete, the content of ellagic acid is 1.2 mg/l [31]. Grape stems can also be a valuable source of such a biologically active substance from the group of stilbene compounds as *trans*-resveratrol [32].

According to D. Harman, the founder of the free radical theory of aging, according to which premature aging of the human body occurs as a result of an increase in the amount of damage caused by free radicals, the use of supplements in the daily diet containing substances that exhibit antioxidant properties will not only increase the quality of human life and reduce the rate of accumulation of age-related changes and chronic disability in older people but also reduce mortality from cancer and cardiovascular diseases.

Aged distilled drinks are part of the human diet and are widely consumed throughout the world, so research aimed at increasing their antioxidant properties and containing more biologically active substances is relevant. To achieve this goal, the use of winemaking waste, which, despite the high content of valuable antioxidants in them, is practically not used, is intended to improve the level and quality of human life.

**Figure 1:**
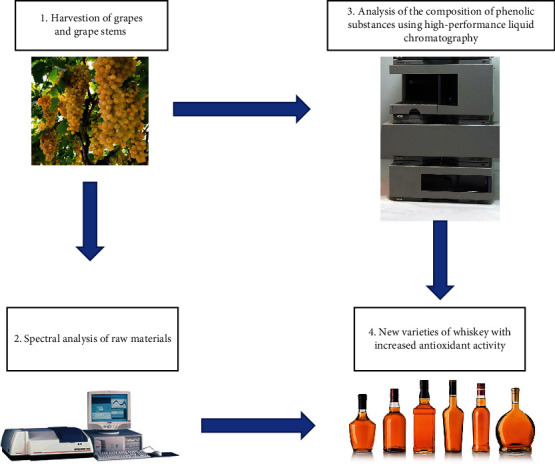
Schematic representation of the whiskey creation with increased AOA due to grape stem extract.

Schematic representation of the whiskey creation with increased AOA due to grape stem extract is presented in Figure 1.

## 2. Materials and Methods

### 2.1. Reagents and Solutions

The following reagents were used for spectrographic analysis: 100 g of sodium tungstate (Na_2_WO_4_·2H_2_O) and 25 g of sodium molybdate (Na_2_MoO_4_·2H_2_O) were dissolved in 700 ml of distilled water. Add 50 ml of concentrated orthophosphoric acid (H_3_PO_4_) 85% (*ρ*20 = 1.71 g/ml) and 100 ml of concentrated hydrochloric acid (HCl) (*ρ*20 = 1.19 g/ml). Bring to a boil and reflux for 10 h. Then, add 150 g of lithium sulfate (Li_2_SO_4_ 2H_2_O) and a few drops of bromine and boil for 15 min. Standard solution of gallic acid: 1.2 g of gallic acid was measured and placed in a flask with a volume of 0.5 l, dissolved in a small amount of an aqueous-alcohol solution of tartaric acid, and the volume of the solution was brought to 0.5 l.

The following reagents were used for chromatographic analysis: solution A—methyl alcohol; solution B—an aqueous solution of trifluoroacetic acid (C_2_HF_3_O_2_) with a mass concentration of 0.6 g/100 ml. The *trans*-resveratrol, (−)-epicatechin, and syringic acid (Sigma-Aldrich) were used as standards.

The following reagents were used for amperometric analysis: eluent—the solution orthophosphoric acid (H_3_PO_4_) with a molar concentration of 2.2 mmol/l. The eluent solution was prepared as follows: Approximately 700 ml of bidistilled water was poured into a 1 l volumetric flask, and 0.15 ml of concentrated H_3_PO_4_ and 10.0 ml of 96% ethyl alcohol (C_2_H_5_OH) were added using a pipette dispenser. The resulting solution was brought to a volume of 1 l with bidistilled water. 2,5,7,8-Tetramethylchroman-2-carboxylic acid (C_14_H_18_O_4_, Trolox-C, Sigma-Aldrich) was used as an antioxidant standard. Preparation of a working solution of Trolox-C with a mass concentration of 100 mg/l: measured out 0.0057 g of Trolox-C and added 30 ml of methyl alcohol; after dissolution, the volume of the Trolox-C solution was brought to 50 ml with the eluent.

### 2.2. Samples

The samples for the research were: 5 samples of Crimean whiskey, aged from 15 to 22 years, produced by Yalta Port LLC and presented in Table 1; alcoholic extracts of grape stems of 11 white grape stems of the *Vitis vinifera* species (Aligote, Rkatsiteli, Colombard, Shabash, Tashly, Soldaiya, Abla, Aurora, Pervenetz Magaracha, Podarok Magaracha, and Kok Pandas) from the ampelographic collection of grapes of the FSBSI Institute Magarach of the RAS; 55 experimental whiskey samples with the addition of 11 prepared alcoholic extracts of grape stems.

The preparation of each of the analyzed whiskey samples involved the use of individual technological regimes and parameters based on varying the duration of aging and the environment (oak species and previous use of oak containers) in which the extraction process took place. Thus, the main distinctive features of the analyzed whiskey samples were as follows:

- DWS Orkney Islands Refill Butt (OIRB): distillate aged for 15 years in a cask previously used for the production of sherry;
- The Orkney Islands Massandra Lacryma Christi Cask (OIMLCC): distillate aged for 15 years in a barrel previously used for the production of white dessert wine Lacryma Christi;
- Orkney Islands Massandra Madera Cask (OIMMC): distillate aged for 16 years in a barrel previously used for the production of Madeira;
- Glentauchers Crimean Brandy/Livadiya Red Port Cask (GCB/LRPC): distillate aged for 22 years in a barrel in which Crimean brandy and Livadiya red port were subsequently aged;
- Tobermory Fill Allier Cask (TFAC): distillate aged for 22 years in a barrel made from Allier oak.

As a source of biologically active substances and antioxidants, an alcoholic (70%) extract of the stems of white technical grape varieties of the Vitis vinifera species was analyzed and used to develop a new type of product based on blending with whiskey.

### 2.3. Equipment

The mass concentration of phenolic substances was identified using a Specord 40 Analytik single-beam scanning spectrophotometer Jena with a working wavelength range of 190–1100 nm.

The composition of phenolic substances was analyzed by high-performance liquid chromatography (HPLC) using an Agilent Technologies chromatography system (model 1100) with a diode array detector. For separation, we used a Zorbax SB-C18 chromatographic column with a size of 2.1 × 150 mm, filled with silica gel grafted with an octadecylsilyl phase with a sorbent particle size of 3.5 *μ*m.

Measurements of the content of water-soluble antioxidants (based on total antioxidant capacity) were carried out using the amperometric method (AOA_am_) on an amperometric flow analyzer with special software for collecting and processing data “Tsvet Yauza-01-AA,” operating within a potential range from +2.0 to – 2.0 V. An electrode made from glassy carbon, which is most suitable for the determination of phenolic substances, was used as a working electrode.

### 2.4. Methodology for Preparing Stem Extracts and Estimating Antioxidant Capacity

In the process of preparing an alcoholic extract of grape stems, the conditions for the preparation of grape stems were established: The optimal ratio of extractant and stems was determined; the concentration of ethyl alcohol that ensures the highest degree of transfer of phenolic substances into the extract; conditions for optimizing the extraction process.

Thus, it was found that the following technological conditions and parameters are effective for obtaining alcoholic extracts of grape stems: preliminary grinding of grape stems to a particle size of 1–3 cm and drying to a relative humidity level not higher than 15% [33]; extraction of crushed stems with a water–ethanol extractant with a volume fraction of ethyl alcohol of 70% at a “solid phase: liquid” ratio of 1 : 3; ultrasonic treatment [34–36] with an oscillation frequency of 35 kHz until equilibrium concentrations of phenolic substances are established.

The method with the Folin–Ciocalteu reagent is based on the ability of phenolic substances, when oxidized, to reduce phosphotungstic and phosphomolybdic acids, which are part of the Folin-Ciocalteu reagent, to their reduced forms. The resulting tungsten oxide (W_8_O_23_) and molybdenum oxide (Mo_8_O_23_) are colored blue, the intensity of which is measured colorimetrically, and the resulting values are proportional to the mass concentration of phenolic substances. Measurements were made in quartz cells with an optical path length of 1 cm, in automatic mode. The calibration graph was based on calibration solutions prepared by adding to 9 volumetric flasks with a volume of 100 ml—2.5; 5.0; 7.5; 10.0; 12.5; 15.0; 17.5; 20.0; 25.0 ml of a standard solution of gallic acid and bringing them to a volume of 100 ml with an aqueous alcoholic solution of tartaric acid. A calibration graph was constructed based on the obtained absorption values. Based on the obtained absorption values, a calibration graph was constructed. The arithmetic mean of the results of two parallel measurements was taken as the final result.

The qualitative and quantitative composition of phenolic substances in the test samples was determined by HPLC using an Agilent Technologies chromatographic system (model 1100) with a diode array detector; chromatography was carried out in gradient mode. During the chromatography process, the composition of the eluent underwent changes in the content of component B, according to the following scheme: 0 min 8%; 0–8 min, 8–38%; 8–24 min. 38–100%; 24–30 min 100%. Eluent flow rate: 0.25 ml/min.

Chromatograms were recorded at the following wavelengths:

- 280 nm: gallic acid, (+)-D-catechin, (−)-epicatechin, and procyanidins;
- 313 nm: derivatives of hydroxycinnamic acids;
- 371 nm: quercetin.

Individual compounds were identified by comparing their spectral characteristics with the spectra described in the literature and by matching the retention time of the detected peak and the peak of the standard sample. The spectral characteristics of individual substances were confirmed using literature data. Calculation of the quantitative content of individual components was carried out using calibration graphs of the dependence of the peak area on the concentration of the substance, constructed using solutions of standard substances. All determinations were carried out in triplicate.

Detection of antioxidant capacity by the amperometric method consisted of determining the strength of the electric current arising during the oxidation of the test substance on the surface of the working electrode at a certain potential and comparing the resulting signal with the signal from the antioxidant standard under the same measurement conditions.

In the process of measuring antioxidant capacity, the eluent feed rate was 1.2 ml/min. To construct the calibration graph, the potential was set at +1.3 V. Calibration curves were constructed by measuring solutions of the Trolox-C standard with a mass concentration of 0.2, 0.5, 1.0, and 4.0 mg/l; for this purpose, 0.02 was introduced into volumetric flasks with a volume of 10 ml: 0.05, 0.1, and 0.4 ml of Trolox-C solution and brought to a volume of 10 ml with eluent. The calibration characteristic of the analyzer is established in the form of a linear dependence of the arithmetic average values of the output signal on the mass concentration of the Trolox-C standard. The calibration characteristic is considered acceptable in the case when the correlation value is not lower than 0.99, and the confidence probability value is 0.95. The magnitude of the analyzer's output signal is determined as a result of five consecutive measurements for each parallel prepared sample of the analyzed sample. The result is taken as the arithmetic mean of 5 measurements; the standard deviation should not exceed 5%.

### 2.5. Data Processing

The mass concentration of phenolic substances in grape stem extracts, whiskey, and model whiskey solutions with stem extracts was measured spectrophotometrically using WinASPECT® software. The concentration of natural phenolic substances was carried out by HPLC using Agilent 1100 software. The results of regression analysis were used for evaluation. Microsoft Excel 2016 was used for mathematical operations.

## 3. Results and Discussion

### 3.1. Phenolic Composition of Whiskey Aged From 15 to 22 Years

The most widely used whiskey technology is the use of oak barrels in which the sherry preparation process was carried out. Thus, we used OIRB whiskey as a control sample, the production of which involved aging the distillate in a barrel previously used for the production of sherry.

Hydroxybenzoic acids, catechins, and flavonols were identified in the phenolic composition of whiskey (Table 2).

The phenolic composition of whiskey in the presented samples was determined, first of all, by the extraction conditions, which largely depended on the conditions, duration of aging, and structural characteristics of the wood of the oak barrel in which aging was carried out.

According to the data in Table 2, the highest content of monomeric forms of phenolic substances according to HPLC and the highest mass concentration of phenolic substances according to Folin–Ciocalteu was characterized by a sample of GCB/LRPC whiskey, in which these values were 157.9 mg/l and 624.0 mg/l, respectively, and the lowest were 65.9 mg/l and 282.0 mg/l of OIMLCC whiskey, which is apparently due to differences in the duration of aging and the characteristics of the previous use of oak barrel Glentauchers whiskey. Crimean Brandy/Livadiya Red Port Cask also found the highest content of hydroxybenzoic acids and procyanidins, compared to other samples, amounting to 61.8 mg/l and 92.1 mg/l.

The highest content of hydroxycinnamic acids, catechins, and flavonols was found in OIMMC whiskey and amounted to 2.0 mg/l, 7.1 mg/l, and 1.7 mg/l, respectively.

**Figure 2:**
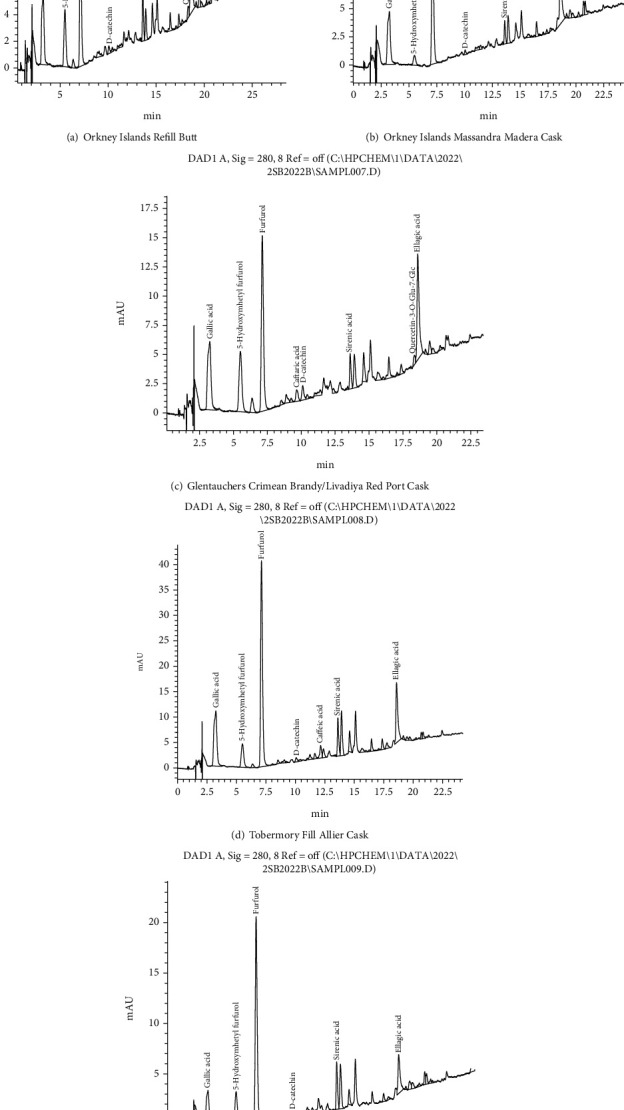
Chromatograms of whiskey.

The component phenolic composition of whiskey aged 15 years, determined by HPLC, is presented in Figures 2(a) and 2(b).

From the chromatograms presented in Figure 2, it is clear that whiskeys of the same aging period have a fairly similar composition, which is also confirmed by the data in Table 1, in particular, whiskey brands OIRB and OIMLCC have similar mass concentration values of the main identified monomers forms of phenolic substances, amounting to 31.2 and 39.6 mg/l.

According to the chromatogram (Figure 2(c)) and data in Table 2, in OIMMC whiskey aged 16 years, compared to whiskey aged 15 years (Figures 2(a) and 2(b)), all identified monomeric forms of phenolic substances are present in large quantities, and their total amount was 51.1 mg/l. The phenolic composition of whiskey aged 22 years is presented in Figures 2(e) and 2(d).

Whiskeys with the longest aging period of 22 years from the analyzed samples, according to their phenolic composition, are superior to less aged samples, both in terms of the mass concentration of phenolic substances according to Folin–Ciocalteu (571.0 mg/l and 624.0 mg/l) and in the total content of monomeric forms of phenolic substances amounted to 64.7 mg/l and 65.8 mg/l.

### 3.2. Phenolic Composition of Aqueous-Ethanol Extracts of Grape Stems and Products Using Them

The qualitative and quantitative phenolic compositions of the produced aqueous-ethanol extracts of grape stems, identified by HPLC, are presented in Table 3.

According to Table 3, the identified phenolic composition of aqueous-ethanol extracts of stems included 6 groups of phenolic substances (comprising 22 phenolic substances) arranged in the following sequence by decreasing their mass concentration: procyanidins > catechins > hydroxycinnamic acids > flavonols > hydroxybenzoic acids > stilbenes. Separately, it should be noted the content of valuable biologically active substances—stilbenes, which belong to the natural phytoalexins of grapes, the content of which, according to our data, reaches a value of 31.2 mg/l.

**Figure 3:**
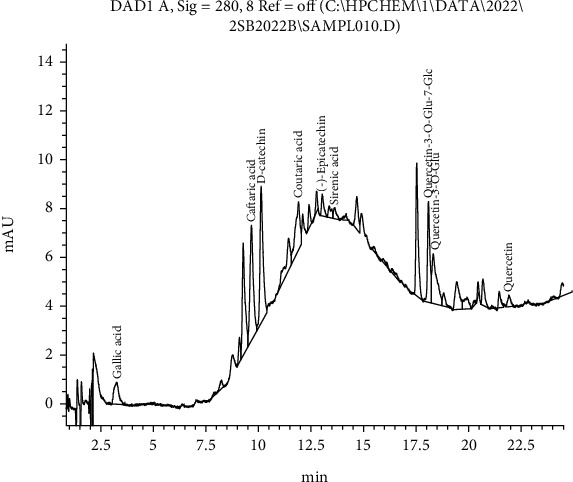
Chromatogram of the developed type of product based on whiskey and alcoholic extract of stems.

The developed type of product based on whiskey and alcoholic extract of grape stems is presented in Figure 3.

Developed on the basis of whiskey and alcoholic extract of grape stems, it contains phenolic substances that exhibit high antioxidant properties, such as (+)-D-catechin, gallic acid, ellagic acid, and *trans*-resveratrol.

### 3.3. Antioxidant Capacity of Whiskey and a Prototype Product Using Alcoholic Extracts of Stems

The values of the antioxidant capacity and mass concentration of phenolic substances according to Folin-Ciocalteu of the analyzed whiskey samples and the developed type of product based on OIRB brand whiskey and alcoholic extract of grape stems are presented in Table 4.

According to Table 4, the mass concentration of phenolic substances in whiskey aged from 15 to 22 years ranged from 0.282 to 0.624 g/l, while the lowest and highest values were characteristic of whiskey samples aged 22 years. The mass concentration of phenolic substances in prototypes prepared from whiskey and alcoholic extracts of grape stems varied from 0.395 to 0.738 g/l, which on average exceeded the value of this indicator compared to the control by 1.3 times.

As follows from Table 4, the value of the antioxidant capacity of whiskey aged from 15 to 22 years is in the range from 0.510 to 0.558 g/l, while the value of the antioxidant capacity practically did not change with changes in the aging period. The value of antioxidant capacity in prototypes based on whiskey and alcoholic extracts of grape stems varied in the range from 0.579 to 0.690 g/l, which on average exceeded the level of antioxidant capacity compared to the control by 1.2 times. The prototype prepared from OIRB whiskey and alcoholic extract of Rkatsiteli grapes was characterized by the highest antioxidant capacity −0.690 g/l.

## 4. Conclusions

Currently, the issue of whiskey production in the Russian Federation is receiving significant attention, while the production of the alcoholic drink itself includes the use of a wide range of production technology schemes, which affects the qualitative and quantitative phenolic composition and, as a consequence, the antioxidant capacity exhibited by the drink. During the study, samples of whiskey with increased antioxidant capacity were obtained based on the use of alcoholic extracts of grape stems of the *Vitis vinifera* species.

The addition of an antioxidant additive to whiskey by blending—an alcoholic extract of grape stems—made it possible to significantly increase the value of the antioxidant capacity of the finished product by 27.7%, the mass concentration of catechins by 17.3%, hydroxybenzoic acids by 15.1%, and the content of *trans*-resveratrol to a value of 4.9 mg/l, despite the fact that there is no trans-resveratrol in whiskey prepared in the traditional way.

Grape stems, which are a valuable source of natural antioxidants, when extracted from grape stems and obtained from them into new types of products, can significantly increase the antioxidant capacity of whiskey by 1.3 times and introduce new components into this type of drink that can increase the immune status of the human body and have a beneficial effect on the antioxidant system of the human body as a whole.

## Figures and Tables

**Table 1:** Name of whiskey samples produced by Port-Yalta LLC.

**No.**	**Name**	**Volume fraction of ethyl alcohol (% vol.)**	**Aging period (year)**	**Number of bottles**
1	Orkney Islands Refill Butt	55.3	15	181
2	Orkney Islands Massandra Lacryma Christi Cask	53.6	15	250
3	Orkney Islands Massandra Madera Cask	53.9	16	230
4	Glentauchers Crimean Brandy/Livadiya Red Port Cask	47.1	22	72
5	Tobermory Fill Allier Cask	56.5	22	231

**Table 2:** Phenolic composition of whiskey identified by HPLC.

**Mass concentration (mg/l)**	**OIRB**	**OIMLCC**	**OIMMC**	**GCB/LRPC**	**TFAC**
Hydroxybenzoic acids	36.3	31.0	40.3	61.8	52.9
Hydroxycinnamic acids	—	—	2.0	1.1	—
Catechins	2.2	0.2	7.1	2.9	1.8
Flavonols	1.2	—	1.7	—	—
Procyanidins	67.6	34.7	28.9	92.1	73.1
MC_ps_ by HPLC	107.2	65.9	80.0	157.9	87.9
MC_ps_ according to Folin–Ciocalteu	542.0	282.0	324.0	624.0	571.0
MC_mfps_ by HPLC	39.6	31.2	51.1	65.8	64.7

*Note:* MC_ps_ by HPLC—mass concentration of phenolic substances by HPLC (mg/l); MC_ps_ according to Folin–Ciocalteu—mass concentration of phenolic substances according to Folin–Ciocalteu (mg/l); MC_mfps_ by HPLC—mass concentration of monomeric forms of phenolic substances (mg/l).

**Table 3:** Qualitative and quantitative phenolic compositions of alcoholic extracts of the stems of white grape varieties of the *Vitis vinifera* species.

**Mass concentration (MC)**	**Indicator value (min ÷ max)/(avg.) (mg/l)**
MC of hydroxycinnamic acids and their esters: caftaric acid, ellagic acid, koutaric acid, caffeic acid, fertaric acid, *p*-coumaric acid, and *p*-coumaric acid ethyl ester	$\frac{58.5-270.8}{163.2}$
MC of hydroxybenzoic acids: gallic acid, syringic acid, and protocatechuic acid	$\frac{65.7-186.3}{93.6}$
MC catechins: (+)-D-catechin and (−)-epicatechin	$\frac{209.4-350.6}{303.6}$
MC of flavonols: quercetin, quercetin-3-O-glucuronide, quercetin-3-O-glucoside-7-O-glucuronide, quercetin-3-O-glucoside, isorhamnetin-3-O-glucoside, kaempferol-3,7-di-O-glucoside, and kaempferol	$\frac{50.4-217.1}{97.5}$
MC stilbenes: *ε*-viniferine and *trans*-resveratrol	$\frac{25.8-31.2}{28.7}$
MC of procyanidins	$\frac{6414.0-8303.0}{6745.0}$

**Table 4:** Mass concentration of phenolic substances and antioxidant capacity of production samples of whiskey of Port-Yalta LLC and prototype products.

**No.**	**Name**	**MC **_**ps**_ **according to Folin–Ciocalteu (g/l)**	
		**Control**	**Prototype**
1	Orkney Islands Refill Butt (OIRB)	0.421 ± 0.006	0.558 ± 0.020
2	Orkney Islands Massandra Lacryma Christi Cask (OIMLCC)	0.324 ± 0.015	0.431 ± 0.017
3	Orkney Islands Massandra Madera Cask (OIMMC)	0.542 ± 0.002	0.616 ± 0.003
4	Glentauchers Crimean Brandy/Livadiya Red Port Cask (GCB/LRPC)	0.624 ± 0.002	0.738 ± 0.025
5	Tobermory Fill Allier Cask (TFAC)	0.282 ± 0.005	0.395 ± 0.015

*Note:* Control—production sample of whiskey. The prototype is based on a production sample of whiskey and an alcoholic extract of grape stems.

## Data Availability

Data is available on request from the authors.
